# Lymphangioma Cavernosum with a Rare Location

**DOI:** 10.3390/jcm11061643

**Published:** 2022-03-16

**Authors:** Justyna Smaga, Oskar Kornasiewicz, Bogna Ziarkiewicz-Wróblewska, Joanna Podgórska, Piotr Krawczyk, Michał Grąt, Marek Krawczyk

**Affiliations:** 1 Department of General, Transplant and Liver Surgery, Medical University of Warsaw, 02-097 Warsaw, Poland; jsmaga@o2.pl (J.S.); oskar.kornasiewicz@wum.edu.pl (O.K.); piotrek.m.krawczyk@gmail.com (P.K.); michal.grat@gmail.com (M.G.); 2 Department of Pathology, Medical University of Warsaw, 02-097 Warsaw, Poland; wroblewskabogna@o2.pl; 3 2nd Department of Clinical Radiology, Medical University of Warsaw, 02-097 Warsaw, Poland; joanna.podgorska@wum.edu.pl

**Keywords:** lymphangioma cavernosum, rare abdominal tumour, surgery, histopathology, diagnostic procedure

## Abstract

Lympangioma cavernosum in the abdominal cavity is a rare benign tumour. In most cases, such tumours are diagnosed in the oral cavity and neck. The aim of this paper is to present our clinical observations and review of existing literature to draw attention to this disease. A 25-year-old woman was admitted to our department for a symptomatic tumour in the lesser curvature of the stomach. The patient was first operated on as a three-year-old child because the tumour extended from the back wall of the stomach to the gastro-colonic ligament. The medical records showed that it was a benign tumour; however, no diagnosis was specified. No symptoms were observed for 22 years. Currently, the patient is admitted for epigastric pain. Abdominal computed tomography revealed an extensive mass located between the left segments of the liver and the lesser curvature of the stomach. The patient was operated on, and the tumour was completely removed. The patient’s postoperative course was uneventful. Histopathological examination of the lymphangioma cavernosum was performed. Two months after the operation, the patient did not report any complaints.

## 1. Introduction

The first descriptions of lymphangiomas were presented in the literature by Redenbacker in 1828 [1] and Guttmann in 1890 [2], focusing on changes in the oral cavity and neck. In 1913, Gaudier and Gorse discussed the histological features of lymphatic cavernous haemangiomas [3]. Cavernous lymphangioma is composed of lymph vessels covered with an endothelial monolayer containing lymph or serous fluid. Most lymphangiomas (80%) are diagnosed below the age of five, while the remaining patients comprise of all ages; however, most often occur around 40 years old [4,5]. Lymphangiomas are rare benign tumours. Most often, these are congenital developmental disorders of the lymphatic system. However, the inflammatory process, which leads to impaired lymph flow and causes cystic changes may also affect the lymphatic vessels [6,7,8,9].

In terms of the histopathological structure, lymphangiomas can occur as:

1. Capillary hemangiomas (Lymphangioma simplex).
2. Cystic lymphangioma (Cystic hygroma).
3. Cavernous lymphangioma.

Hemolymphangiomas, which are another type of lymphangioma, are composed of both lymphatic and blood vessels [10].

The first type of lymphangioma (Lymphangioma simplex), also known as capillary lymphangioma, usually occurs in the oral cavity, specifically the mandible; but it can also occur on the trunk and limbs. Moreover, it affects the skin and subcutaneous tissue and is diagnosed mainly in neonates and older children [11,12].

The second type of lymphangioma (cystic hygroma or cystic lymphangioma) most often develops in the neck [13].

Cavernous lymphangiomas are rare lesions found in the mediastinum and sometimes in the abdominal cavity [14]. Within the abdominal cavity, they are most often located in the mesentery, net, and retroperitoneum. They constitute < 5% of all lymphangiomas, and are more commonly located in different parts of the head, face, or neck [15]. They may also occur in the walls of the small bowel or stomach, most often in the submucosa [16,17,18,19,20,21,22].

So far, only a few cases of lymphangioma in the lesser curvature of the stomach have been described in existing literature. The rarity of lymphangioma in the abdominal cavity, especially close to the stomach, prompted us to present our case and review of the literature to draw attention to this disease during the differential diagnosis for patients.

In some cases, endoscopic examination is helpful, while endoscopic ultrasound (EUS) examination is the decisive factor sometimes [17].

In the clinical course, it is necessary to distinguish cavernous lymphangioma of the gastrointestinal tract from other cystic tumours, mainly vascular, and from lymphatic vessel dilatations (lymphangiectasia). The latter can develop from inflammatory processes, but also due to blockage by a neoplastic tissue, requiring clinical explanation. These changes are usually asymptomatic and diagnosed incidentally [23].

## 2. Clinical Observation

A 25-year-old female patient was referred to the Department of General, Transplantation, and Liver Surgery at the Medical University of Warsaw due to a symptomatic tumour in the lesser curvature of the stomach. The patient complained of epigastric pain and pressure, especially after meals.

At three years old, the patient was hospitalised in the Paediatrics Surgical Department due to peritoneal symptoms and radiological signs of impaired gastrointestinal patency. The patient underwent emergency surgery where an enormous, soft lesion extending from the back wall of the stomach to the gastro-colonic ligament was found intraoperatively. According to the operative description, the tumour was completely removed. In the postoperative period, a lymphatic outflow through the drain from the peritoneal cavity was observed up to the 20th day of hospitalisation. Moreover, on the fifth postoperative day, there was bleeding in the operated area, which manifested as the outflow of blood through the drain. This complication was treated conservatively. The child was discharged in good general condition on the 25th day of hospitalisation. The medical records from that period stated that it was a benign tumour, without specifying the diagnosis. 

The patient’s current symptoms occurred three months prior to admission to our department. Due to abdominal pain, gastroscopy was performed, revealing a spherical bulge, approximately three cm in diameter, with no mucosal changes. There was a slight thickening of the mucosal folds. The lesion was soft and susceptible to pressure using an endoscope. The lesion was located along the lower region of the lesser curvature. The gastric specimens collected for histopathological examination revealed chronic mucosal inflammation only with no signs of metaplasia.

**Figure 1:**
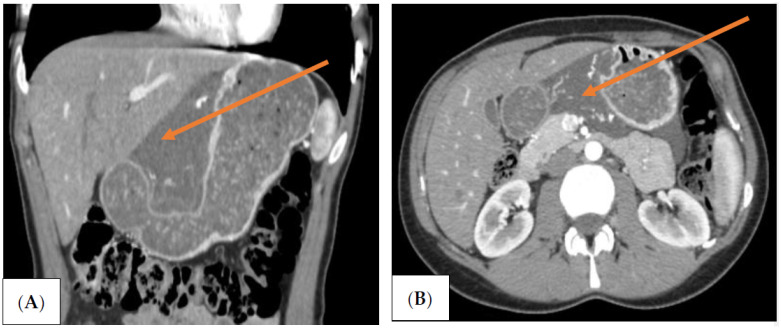
(**A**,**B**) Computed tomography (CT) image of the abdominal cavity. There is a visible mass located at the lesser curvature of the stomach (orange arrows).

Three-phase computed tomography (CT) scan revealed an extensive mass measuring 10 × 4 cm between the left segments of the liver and the lesser curvature of the stomach. The lesion was well-defined, fluid dense, and did not contain solid elements. Within it, there were vessels with an undistorted course. The mass occupied the space in front of the body and tail of the pancreas, descending further down the back of the stomach and duodenum. (Figure 1A,B).

**Figure 2:**
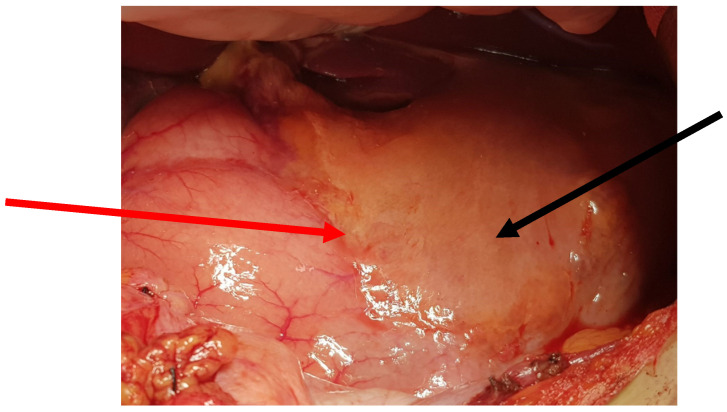
Intraoperative image—The tumour was close to the lesser curvature of the stomach. The arrows indicate: the lesser curvature of the stomach (red arrow), and the anterior surface of the tumour (black arrow).

**Figure 3:**
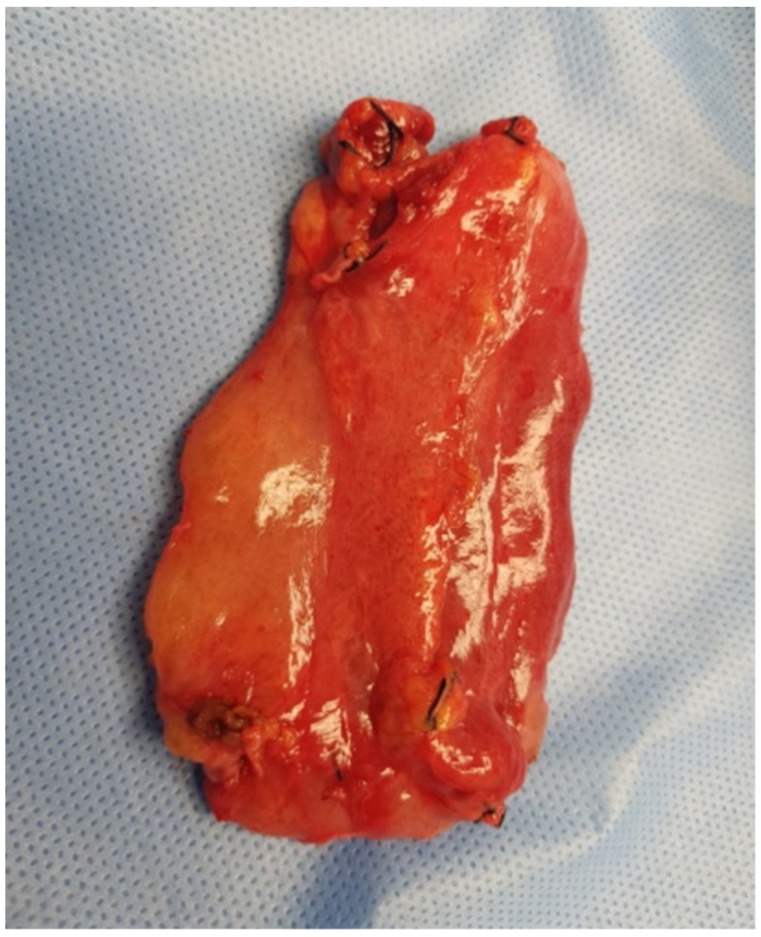
The excised specimen following the operation. The size of the specimen is 7 × 5 cm.

**Figure 4:**
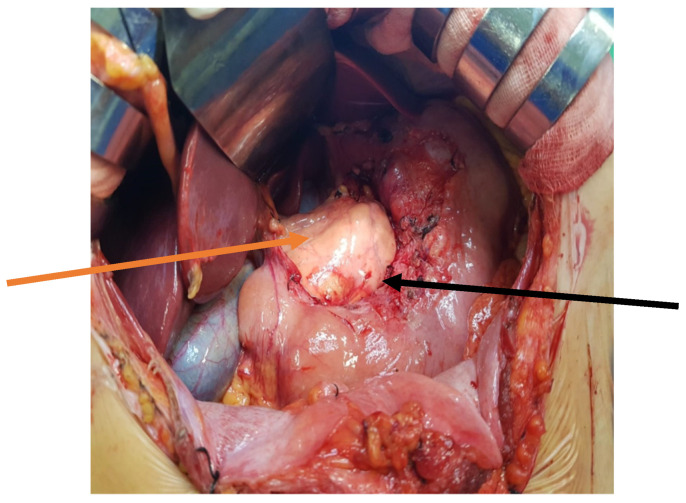
Intraoperative view after tumou r resection of the lesser curvature of the stomach. The arrows indicate the lesser curvature of the stomach (black arrow), and the front surface of the pancreas (orange arrow).

The patient was qualified for elective surgery. The presence of an approximately seven-cm long soft tissue extending along the lesser curvature of the stomach, closely connected to the stomach wall, was found intraoperatively (Figure 2). The tumour penetrated the gastrohepatic ligament, but did not reach the liver. The lesion did not penetrate the retroperitoneal space or infiltrate the pancreas. The tumour was completely removed without gastric wall resection (Figure 3). After removal of the lesion, the serosa of the lesser curvature of the stomach was secured with a continuous PDS 4/0 suture (Figure 4).

The patient’s postoperative course was uneventful. The patient, with a healed wound, was discharged on the 10th day of hospitalisation. Two months after the operation, during the control visit, the patient did not report any complaints. 

**Figure 5:**
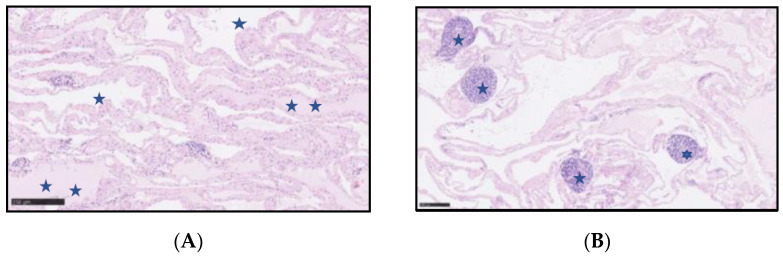
(**A**) Lymphangioma cavernosum—Vascular spaces (one blue star), some of them with a proteinaceous fluid (two blue stars). (**B**). Lymphangioma cavernosum—Small clusters of lymphocytes (one blue star) within the lymphangioma. Haematoxylin and eosin staining.

**Figure 6:**
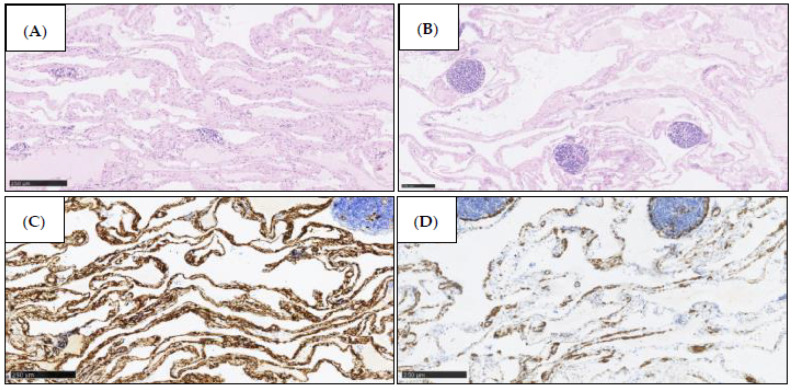
Positive immunohistochemical stain for D2-40 (**A**), CD31 (**B**), CD34 (**C**) in the vascular endothelium. SMA positive in myofibroblasts of connective tissue septum (**D**).

On gross examination, the resected specimen had a non-encapsulated, spongy, focally gelatinous mass measuring 7 × 5 × 2 cm. Microscopically, thin-walled, dilated vessels with different sizes, lined by a flattened endothelium without atypia (Figure 5A,B), and predominantly lymphatic in origin were observed (D2-40+, CD31+, CD34+, factor VIII+) (Figure 6A–C). Their lumina mostly contained proteinaceous fluid, with occasional admixture of lymphocytes, both B (CD20+) and T (CD3+) cells, histiocytes (CD68 KP1+), and erythrocytes, without plasma cells (CD138−, IgG4−). Larger lymphatic vessels with a layer of smooth muscle were also visible. Small lymph nodes with a preserved architecture (CD20, CD3, BCL6, CD23) and sinus dilatation were prevalent, as well as numerous smaller clusters of B and T lymphocytes (Figure 5B). Focal interstitial fibrosis and thickening of the connective tissue septum with myofibroblast proliferation were observed (SMA) (Figure 6D). The tumour focally surrounded normal blood vessels and nerve bundles and penetrated the adjacent adipose tissue, but not the stomach wall. The patient was diagnosed with a lymphangioma cavernosum.

## 3. Discussion

Most abdominal lymphangiomas (1% of all lymphangiomas) are cystic in nature and are in the mesentery and retroperitoneal space [5,8,9]. Lymphangiomas in the stomach are rare. To date, 12 cases of gastric lymphangiomas have been described in the English literature and 23 cases in Japanese reports [3,4,5,6,7,17]. In a few cases, the tumour was in the gastric submucosa. In these patients, gastroscopy, especially EUS, is very helpful for diagnosis. In such situations, it is possible to successfully excise the lesion using endoscopic techniques [17].

In another patient, a tumour in the gastric wall was removed during gastrectomy [24].

According to current literature, the mean age of patients with gastric lymphangiomas is 47 years old (5–79 years). Haemangiomas occurred at a similar frequency in both men and women. In most cases, tumours were found accidentally during surgery for other reasons, and the patients did not report symptoms directly related to the tumour.

These tumours manifest clinically at the end of the second year of life and, similar to our patient, depend on the location [5]. These include nausea, vomiting, stomach pain, weight loss, or signs of gastrointestinal obstruction. Most patients see their doctor when the chronic symptoms are exacerbated from the pressure exerted by the growing tumour on the surrounding tissues. However, these changes occur asymptomatically or with very few symptoms, and their presence is found accidentally.

Therefore, making a correct diagnosis before surgery is difficult because of the non-specific symptoms with varying severity. To properly plan surgical treatment, it is necessary to perform sonography, CT, or magnetic resonance imaging (MRI) of the abdominal cavity to obtain information about the nature of the haemangioma, its location, size, and involvement of adjacent organs. Within the abdominal cavity, lymphangiomas are most often located within the mesentery or retroperitoneal space, although localisation within organs or the abdominal wall is also possible. In sonography, these are anechoic structures, possibly containing small areas of debris. CT and MRI examinations with contrast agents usually present as cystic, fluid-filled structures, often with a thickened wall and septum. Sometimes, these lesions may show signs of previous bleeding or infection. Moreover, being located within the small intestinal wall may predispose to intussusception [21].

Preoperative imaging tests allow for suspicion of lymphangioma; however, the final diagnosis including the subtype of the lymphangioma is made based on histopathological examination of the postoperative material.

The most common treatment for patients with gastric lymphangioma is operative.

In 2020, a study was published presenting an extensive meta-analysis of the treatment of patients with angiomas, including those in the lymphatic system. It emphasises that a large group of patients with abdominal lymphangiomas do not have symptoms and are detected incidentally. They recommended that this group of patients be subjected to clinical observation, and the decision to treat should be made based on the location of the lesion, its size, whether it tends to increase, and above all, symptoms such as abdominal pain, vomiting, and gastrointestinal obstruction. The authors do not recommend sclerotherapy of lesions located in the abdominal cavity (applied in superficial locations), both due to the recurrence of lesions and the possibility of complications. In symptomatic patients, especially when the lesion in the abdominal cavity becomes enlarged, surgery is recommended, bearing in mind the risk of complications [25].

In the present case, the decision was made to perform an operative treatment on the patient.

The question of whether the lymphangioma is a true neoplastic tumour, a hamartoma, or lymphangiectasia remains a controversial issue [5]. Most researchers consider lymphangioma to be a malformation that develops during the embryonic period when the lymphatic system is formed. According to the first theory, the lymphatic system is derived exclusively from the endothelium of the venous vessels and is formed due to the “budding” of lymphatic vessels from pre-existing embryonic veins. According to the second hypothesis, peripheral lymphatic vessels arise from putative mesenchymal lymphangioblasts, regardless of the venous system. They form lymphatic vessels and then fuse with the veins. The third theory suggests the veno-mesenchymal origin of the lymphatic system: central lymph vessels develop from the venous system, and the peripheral lymph vessels arise by in situ differentiation of mesenchymal precursor cells. This process is regulated by numerous cytokines.

The cause of the haemangioma is incorrect and lacks connections with the venous system or their abnormal budding from the embryonic veins. Lymphangioma can also develop from fragment sequestration of lymphoid tissue and its lack of connection with the lymphatic system [26].

Most lymphangiomas occur in childhood and are located where the primary lymphatic sac develops (primitive limfatic sac), which are the head, neck, and inguinal region. They often increase in proportion to the patient’s age.

Lymphangiomas develop much less frequently in adults, probably due to a minor defect or damage to the lymphatic system, compensated under normal conditions. Factors leading to increased lymphatic volume may lead to haemangiomas [26].

It is possible that some haemangiomas are acquired lesions caused by an obstruction in the lymphatic system, for example, during surgery, trauma, radiotherapy, infection, or chronic inflammation.

The benign nature of lymphangiomas is generally accepted. However, the development of haemangioma may also result from neoplastic proliferation of the endothelium or lymphatic stromal cells secondary to the dysregulation of certain growth factors. Experimental studies in mice with anti-amphiregulin demonstrated the potential therapeutic effect of this drug in patients with lymphangiomas [27].

In most cases, the diagnosis of lymphangioma is difficult for pathologists. The presence of haemorrhages, relatively numerous smooth muscle cells within the wall of the lymphatic vessels, and venous malformation may suggest haemangioma cavernosum. In the latter case, the diagnosis of lymphangioma is supported by the presence of lymphocyte clusters in the stroma and greater regularity of vascular spaces lined with loosely located endothelium.

The differential diagnosis of abdominal lesions may include multicystic mesothelioma, microcystic adenoma of the pancreas, other vascular tumours that may contain dilated vascular spaces (kapsiform hemangioendothelioma, Kaposi sarcoma, Dąbska-type hobnail hemangioendothelioma), and myxoid sarcoma. Sometimes, the picture may suggest an angiomyolipoma or myelolipoma. Immunohistochemical staining is the basis for differential diagnosis in doubtful cases. Typical lymphangioma endothelial cells are positive for D2-40 (podoplanin), PROX1, and CD31. The CD34 reaction is variable, and smooth muscle cells surrounding the cystic spaces show reactivity with SMA.

## 4. Conclusions

Abdominal lymphangioma is a rare benign tumour that is usually asymptomatic and detected incidentally. Sonography (sometimes EUS), CT, or MRI with contrast are helpful in the preoperative diagnosis, although they can also provide an unambiguous diagnosis. Therefore, surgery is recommended. The final diagnosis was made via histopathological examination of the postoperative material using immunohistochemical staining.

## Data Availability

The data is available from the Authors upon reasonable request.
